# Emerging Technologies for the Production of Renewable Liquid Transport Fuels from Biomass Sources Enriched in Plant Cell Walls

**DOI:** 10.3389/fpls.2016.01854

**Published:** 2016-12-08

**Authors:** Hwei-Ting Tan, Kendall R. Corbin, Geoffrey B. Fincher

**Affiliations:** ^1^Centre for Tropical Crops and Biocommodities, Queensland University of Technology, BrisbaneQLD, Australia; ^2^Centre for Marine Bioproducts Development, School of Medicine, Flinders University, Bedford ParkSA, Australia; ^3^Australian Research Council Centre of Excellence in Plant Cell Walls, School of Agriculture, Food and Wine, University of Adelaide, Glen OsmondSA, Australia

**Keywords:** bioethanol, fermentation, hydrothermal liquefaction, lignocellulosic biomass, pre-treatments

## Abstract

Plant cell walls are composed predominantly of cellulose, a range of non-cellulosic polysaccharides and lignin. The walls account for a large proportion not only of crop residues such as wheat straw and sugarcane bagasse, but also of residues of the timber industry and specialist grasses and other plants being grown specifically for biofuel production. The polysaccharide components of plant cell walls have long been recognized as an extraordinarily large source of fermentable sugars that might be used for the production of bioethanol and other renewable liquid transport fuels. Estimates place annual plant cellulose production from captured light energy in the order of hundreds of billions of tons. Lignin is synthesized in the same order of magnitude and, as a very large polymer of phenylpropanoid residues, lignin is also an abundant, high energy macromolecule. However, one of the major functions of these cell wall constituents in plants is to provide the extreme tensile and compressive strengths that enable plants to resist the forces of gravity and a broad range of other mechanical forces. Over millions of years these wall constituents have evolved under natural selection to generate extremely tough and resilient biomaterials. The rapid degradation of these tough cell wall composites to fermentable sugars is therefore a difficult task and has significantly slowed the development of a viable lignocellulose-based biofuels industry. However, good progress has been made in overcoming this so-called recalcitrance of lignocellulosic feedstocks for the biofuels industry, through modifications to the lignocellulose itself, innovative pre-treatments of the biomass, improved enzymes and the development of superior yeasts and other microorganisms for the fermentation process. Nevertheless, it has been argued that bioethanol might not be the best or only biofuel that can be generated from lignocellulosic biomass sources and that hydrocarbons with intrinsically higher energy densities might be produced using emerging and continuous flow systems that are capable of converting a broad range of plant and other biomasses to bio-oils through so-called ‘agnostic’ technologies such as hydrothermal liquefaction. Continued attention to regulatory frameworks and ongoing government support will be required for the next phase of development of internationally viable biofuels industries.

## Introduction

The demand for chemical energy is projected to increase by 0.7-1.2% annually for the next 20 years, in which 64% of the total increase in demand will be attributable to the transportation sector (Bp Energy Outlook 2035, 2015). This increase in global liquid demands is equivalent to a rise of liquid fuel consumption (i.e., oil and biofuels) to 111 million barrels per day (Mb/d) (Bp Energy Outlook 2035, 2015). Energy stored in plant biomass is a potentially renewable material that could contribute substantially to transportation fuel needs at costs competitive with fossil fuel (Somerville, 2006). Plant biomass can be produced in abundance, cheaply and is not geospatially restricted to one biome. There have been several estimates of the annual global production of key components of these plant biomass sources; this production is enabled by the ability of plants to capture solar energy and convert it to chemical energy. It has been calculated that 100 billion tons of land biomass (organic dry matter) and 50 billion tons of aquatic biomass are produced per year (Naik et al., 2010). Of the plant biomass, agricultural, industrial and forest derived lignocellulosic residues are regarded as the largest source of carbohydrate available for making chemical fuels (Jørgensen et al., 2007). Based on these figures, it might be expected that annual production of cellulose could be considerably more than 50 billion tons and lignin more than 10 billion tons per annum. However, to-date the carbohydrates partitioned in plant biomass through photosynthesis are being underutilized; it is estimated that only 2% of this resource is currently used for biofuel production (Pauly and Keegstra, 2008).

The major constituents of food crop residues, specialist biofuel crops, and other plant residues used as biomass sources for biofuel production are cellulose and lignin. These are important components of the extracellular cell wall of plant cells, which provide the strength to support plant structural requirements, together with a number of other biological functions. Cellulose is generally embedded in a lignin matrix and the two wall macromolecules provide the plant structure with enormous tensile and compressive strength, respectively. An obvious consequence of the evolution of tough lignocellulosic cell walls to provide structural strength to land plants, many of which are very large and heavy, is that lignin and cellulose are tightly interwoven into a strong and compact biocomposite that is also resistant to the penetration of degrading enzymes or microorganisms. This resistance to degradation is readily seen through the length of time required for a tree rotting on the forest floor to be completely degraded, and is known in the biofuel industry as ‘recalcitrance’. But the benefits of coming up with strategies to overcome recalcitrance are enormous. Cellulose is a polysaccharide consisting of thousands of (1,4)-linked β-glucosyl residues and released glucose can be fermented by many microorganism to produce biofuels, including bioethanol. Lignin is a large three-dimensional polymer of phenylpropanoid molecules and, as seen with glucose released from cellulose, is an abundant source of high energy, reduced carbon.

The benefits of lignocellulosic biofuels have been well described (Hill et al., 2006; Tilman et al., 2006; Hill, 2007; Cherubini, 2010; Naik et al., 2010). One of the more important properties of lignocellulose is that it is not readily digested by microorganisms in the human gut, and is therefore not in demand as a human food. This overcomes the food vs. fuel debate that has dogged the development of many first generation bioethanol industries, which use edible starch from biomass such as Zea *mays* (corn) grain as a source of fermentable carbohydrate. Nevertheless, producing cost-competitive cellulosic biofuels is challenging because, as mentioned above, lignocellulosic residues are a complex and entwined mixture of carbohydrates and polyphenol polymers, often with associated protein, that are difficult to separate into discrete, usable components and are difficult to penetrate with enzymes. Hence, to convert this recalcitrant biomass into ethanol, fermentable monosaccharides need to be liberated from the network. The processing methods employed to make the carbohydrates accessible, such as various pre-treatments and subsequent enzyme saccharification, can drastically increase the cost (per liter) of ethanol production (Mosier et al., 2005; Alvira et al., 2010). A recent NREL report calculated the economics for biochemical conversion of a second generation biomass (corn stover) to ethanol using dilute acid pre-treatment, enzymatic hydrolysis and co-fermentation. The findings showed that the breakeven cost for lignocellulosic ethanol was ∼$0.60/liter in which the cost of the feedstock contributed $0.20/liter, enzyme $0.09/liter and non-enzyme conversion $0.29/liter (Humbird et al., 2011). Thus, for ethanol production from lignocellulosic biomass to be cost competitive, the biomass must be sourced cheaply, produced abundantly and require minimal processing to drive down investment costs at all stages of production.

Other external factors, such as the current low fossil fuel price of about US $50 per barrel, has placed considerable pressure on the development of lignocellulosic biofuel industries. Profitable production of cellulosic biofuel with the current technology was predicted to be sustainable when crude oil is above US $100 per barrel and different scenarios of the effects of oil price volatility on cellulosic biofuel profitability have been discussed (Reboredo et al., 2016). As history has shown, oil prices are inherently volatile and, in the longer term, fossil fuels are clearly not sustainable because they are non-renewable. During our efforts to reduce our carbon footprint and to ameliorate the effects of rising atmospheric CO_2_ levels on climate, it is imperative that we aim for and achieve continuous progress in renewable industries.

Here, we will provide a brief update on advances that might contribute positively to the profitability of cellulosic biofuel industries and, in particular, we will discuss (i) plant engineering to tailor for higher cellulosic biomass, (ii) current biofuel policies, (iii) cellulosic biofuel conversion methods and the prospect of emerging technologies.

## Biofuel Feedstocks

There have been many research articles and government reports written on emerging biofuels technologies, including a recent and comprehensive treatise compiled under the auspices of UNESCO (Karp et al., 2015). More specifically, reports on the availability, efficacy and conversion of biomass sources for lignocellulosic and other biofuel production systems include the Billion Ton study in the USA, a Commonwealth Scientific and Industrial Research Organization (CSIRO) report on biomass potential from crop and forest residues in Australia and a public report on feedstock and production capacity to produce sustainable aviation fuel (Qantas Airways Ltd and Australian Renewable Energy Agency, 2013).

The title of the Billion Ton Study in the USA (Somerville, 2006) provides an obvious clue as to the scale at which we are working in the identification and characterization of suitable biofuel feedstocks. Examples of agro-industrial waste products and specialist crops include wheat and barley straw, agave leaves, grape marc, algae, wood chip residue, used cooking oil, palm oil, soybean oil, canola oil and tallow, sunflower oil, switchgrass, poplar, *Miscanthus* and others (Kim and Dale, 2004; Somerville, 2006; Gui et al., 2008; Mata et al., 2010; Sannigrahi et al., 2010; Davis et al., 2011; Corbin et al., 2015a,b; Subagyono et al., 2015).

**FIGURE 1:**
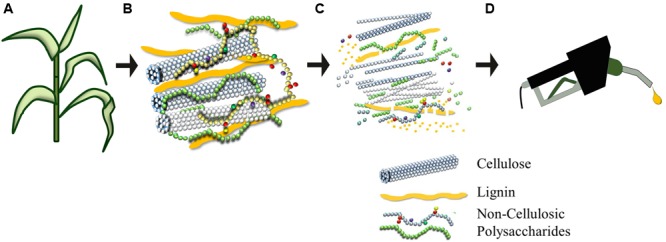
**Plant biomass can be pre-treated to liberate its constituent sugars to produce ethanol **(A–D)**.** Plant cell walls **(B)** consist of an intricate and recalcitrant network that must be degraded for the conversion of the polymeric lignocellulosic biomass to be fermentable precursors for liquid fuel production. Cellulose is a homopolymer of glucosyl units that coalesces to form condensed microfibrils. Non-cellulosic polysaccharides are usually substituted or branched heterogeneous polymers composed of hexoses (glucose and galactose), pentoses (xylose and arabinose), and a wide variety of other sugars. Burton et al. (2010) provide details of the branching of backbone and the diversity of types of non-cellulosic polysaccharide. Lignin is a polymer of phenylpropanoid units that is shown here as elongated units, but which in reality is distributed throughout the cell wall matrix, where it entraps the polysaccharides to create an extremely strong and dense biocomposite that is difficult to degrade.

The compositions of many lignocellulosic feedstocks and waste products have been characterized (**Table 1**). Although informative, the data cannot be used to directly calculate ethanol yields because the specific structures of constituent carbohydrates vary between species and are not indicative of how the material will behave during processing. As noted above, cell wall polysaccharides are held together by covalent and non-covalent linkages and embedded in a network of the non-carbohydrate polymer lignin (**Figure 1**). Cellulose is highly resistant to enzymatic degradation in its own right, but the presence of lignin further reduces access of enzymes to cellulose, which in turn slows the rate and efficiency of hydrolysis (Zeng et al., 2012). In addition, the energy rich, non-food based carbohydrate, cellulose, has been targeted for bioethanol production, but it cannot be directly converted to bioethanol without being firstly hydrolyzed into its monomeric constituent, glucose. The trade-off between input costs and energy required to degrade cellulose into a form that can be converted to ethanol is counterbalanced by its sheer abundance. Cellulose is composed of β-(1,4)-linked glucosyl residues and is the most abundant terrestrial natural biopolymer (Brown, 2004). As the cellulose chains are synthesized, parallel glucan chains aggregate via extensive intermolecular hydrogen bonding and van der Waal forces to form para-crystalline microfibrils (Cosgrove, 2014). When chains do not aggregate in an ordered fashion, amorphous regions are formed (Gomez et al., 2008) and these non-crystalline regions are more accessible for enzymatic hydrolysis. One study has shown that modifications to the relative degree of crystallinity in cellulose significantly influence the rate of its biochemical conversion to the fermentable monosaccharide glucose (Harris et al., 2009).

**Table 1:** Composition of lignocellulosic biomass (% w/w).

	Lignocellulosic biomass	Cellulose	NCP	Lignin	Citations
**Grasses**	*Miscanthus*	38-42	21-23	18-21	Haffner et al., 2013; Kuchelmeister and Bauer, 2015
	Sorghum	15-34	12-18	6-16	Pauly and Keegstra, 2008; Carroll and Somerville, 2009
	Sugarcane	20	10	6	Kuchelmeister and Bauer, 2015
	Switchgrass	33-45	25-35	6-18	Dale et al., 1996; Carroll and Somerville, 2009
	Coastal Bermuda grass	26-32	19-25	15-20	Sun and Cheng, 2005; Wang et al., 2010
	Energy cane	33	23	16	Kuchelmeister and Bauer, 2015
**Woody species**	Beech	43	32	24	Fengel and Wegener, 1983
	Eucalyptus	40-44	10-19	25-37	Inoue et al., 2008; Saxena et al., 2009; Romaní et al., 2010
	Pine	33-43	20-21	27-35	Kuchelmeister and Bauer, 2015
	Poplar	27-37	14-25	21-25	Ibáñez and Bauer, 2014; Kuchelmeister and Bauer, 2015
	Spruce	40		28	Fengel and Wegener, 1983
	Willow	26		23	Kuchelmeister and Bauer, 2015
**Waste**	Barley straw	37-38	26-37	16-19	Sun and Sun, 2002; García-Aparicio et al., 2007
	Corn stover	36-38	28-29	17-21	Yang and Wyman, 2004; Öhgren et al., 2007
	Municipal solid waste	33	9	17	Saxena et al., 2009
	Newspaper	40-62	25-40	18-30	Sun and Cheng, 2002; Saxena et al., 2009
	Rice straw	39-42	20-32	13-14	Zhu et al., 2006; Jin and Chen, 2007
	Rye straw	33	22	20	Sun and Cheng, 2005
	Sunflower stalks	34-39	20-34	17-18	Sharma et al., 2002; Ruiz et al., 2008
	Rapeseed straw	37	24	17	Díaz et al., 2010
	Olive tree prunings	25	16	19	Cara et al., 2008
	Wheat straw	30-39	39-50	15-17	Sun and Cheng, 2002; Sun and Cheng, 2005
	Agave leaves	12-17	9-10	9-13	Corbin et al., 2015a
	Grape marc	5-6	6-11	11-33	Corbin et al., 2015b

*Lignocellulosic biomass is predominantly composed of cellulose, NCP (non-cellulosic polysaccharides) and lignin. The amount and complexity of polymers may be extrapolated to predict ethanol yields. Data are presented as percentage of dry weight (% w/w).*

The remaining carbohydrate components of cell walls that make up lignocellulosic biomass sources consist mainly of a group of heterogeneous polymers known as the non-cellulosic polysaccharides. Non-cellulosic polysaccharides originate from cell walls and represent about 20–35% of lignocellulosic biomass and up to 50% of walls in some cereal grains (Ebringerova and Heinze, 2000; Saha, 2003). These polysaccharides are generally synthesized in the Golgi and exported into the cell wall via secretory vesicles, where they form a gel-like matrix (Lerouxel et al., 2006; Wilson et al., 2006; Burton et al., 2010). Once incorporated into the cell wall, the non-cellulosic polysaccharides become intertwined with the cellulose microfibrils and this adds strength to the wall while maintaining flexibility and porosity (Burton et al., 2010). Compared with cellulose, non-cellulosic polysaccharides have lower degrees of polymerization and are less crystalline, resulting in polymers that are more easily hydrolyzed with dilute acid or enzymes under mild pre-treatment conditions (Lee et al., 1999). Structurally, non-cellulosic cell wall polysaccharides vary widely in amount and composition across the Plant Kingdom and include more common polysaccharides such as xyloglucans, heteroxylans, heteromannans, pectic polysaccharides, and (1,3;1,4)-β-glucans. The polysaccharides are variously composed of monomers that include the pentose sugars (D-xylose and L-arabinose), hexose sugars (mainly D-galactose, D-glucose, and D-mannose), and uronic acids (Lewin and Goldstein, 1992). The efficient utilization of this heterogeneous group of non-cellulosic polysaccharides for bioethanol production is an area of research that is still faced with significant challenges.

To convert lignocellulosic biomass into ethanol, the fermentable monosaccharides need to be liberated from the cell wall matrix. The processing methods employed to make the carbohydrates accessible, such as physical and/or chemical pre-treatments followed by enzymic depolymerization of the polysaccharides to their constituent monosaccharides (saccharification), can drastically increase the cost of ethanol production (Mosier et al., 2005; Alvira et al., 2010). Current calculations suggest that breakdown of lignocellulosic biomass into a form that can be fermented to bioethanol costs twice as much as the depolymerization of corn starch for ethanol production, namely $0.39/liter and $0.21/liter, respectively (Khanna, 2008). The higher price for lignocellulose biomass conversion to monosaccharides is partially attributable to the cost of available feedstocks and handling of the biomass, which on average range from $30-140/MT (Chovau et al., 2013). However, the majority of expenses for lignocellulose conversion are attributable to costly processing methods that are required to degrade the biomass into monosaccharides (Mosier et al., 2005; Alvira et al., 2010). For example, it is estimated that moderate enzyme loadings such as a cellulase dosage of 15 filter paper units, FPU/g cellulose at a commercial scale could correlate to about a 30 g enzyme loading per liter of ethanol produced (Qing et al., 2010), at a cost of $0.03-$0.11/liter (Aden and Foust, 2009; Kazi et al., 2010). However, when saccharification and fermentation costs from corn stover are used in the techno-economic modeling, these costs for enzymes seem to be relatively modest, with a much higher real cost of $0.18-0.39/liter ethanol (Klein-Marcuschamer et al., 2012). As a result, for enzymatic hydrolysis to be economically feasible in large scale productions, the cost of cell wall degrading enzymes must be below $2/kg protein (Qing et al., 2010). The recycling of enzymes for subsequent hydrolysis may be one means to reduce enzyme costs. For example, one study has shown that five batches of pre-treated corn fiber were successfully hydrolyzed using recovered enzyme preparations (Moniruzzaman et al., 1997). Although the recycling of enzymes is favorable, ideally biological or technological advancements will minimize or completely eliminate the need for the application of exogenous enzymes. Another growing area of research that may reduce biomass processing costs is known as consolidated bioprocessing (CBP). This process uses microorganisms that co-produce both enzymes and alcohol during fermentation which eliminates the need for commercial enzymes (Olson et al., 2012).

An over-riding perception with lignocellulosic bioethanol production is that the process is marginal with respect to the mass-energy balance. Predictions of ethanol yields that can be achieved are predominantly based on bench work data (gram scale) that are subsequently extrapolated to predict large scale production (tons scale) under the assumption that scalability is linear. More information is required to validate these assumptions. It might also be argued that the energy density of ethanol is too low, given the relatively high amount of oxygen in the molecule, in comparison to its carbon content.

At this stage many scientific endeavors have been focused on maximizing the ethanol yields that can be achieved from small scale production systems using two approaches, namely tailoring the plant to meet the requirements of the industrial process (Himmel et al., 2007; Coleman et al., 2009; Ambavaram et al., 2011; Harris et al., 2012; Abramson et al., 2013; Puranik et al., 2014) or tailoring the industrial process to a specific feedstock (Adams et al., 2011; Brodeur et al., 2011; Chiaramonti et al., 2012; Menon and Rao, 2012; Mohanram et al., 2013). These approaches will be discussed in the following sections of this review.

## Engineering Plants To Enhance Cellulosic Biomass

For lignocellulosic-based biofuel to become a commercial reality, a multi-faceted approach is required for its conversion. Much progress has been made in process engineering that is used to convert biomass to biofuel by both biochemical and thermochemical methods (Carroll and Somerville, 2009). In addition, the development in plant breeding and biomass modification techniques are important (Carroll and Somerville, 2009), because the provision of suitable and specialist bioenergy crops can greatly reduce costs associated with harvesting, pre-treatment and bioconversion.

Some studies have investigated biological means to reduce the cost of enzyme dosing by altering the cell wall composition or polysaccharide structure of a selected feedstock (Taylor et al., 2008; Mahadevan et al., 2011). For example, transgenic maize plants expressing cell wall degrading enzymes had 141 and 172% higher glucose and xylose yields, respectively, following enzymatic hydrolysis, compared with control plants. The expression of endoglucanase and xylanases in the transgenic maize tissues resulted in a 50% increase in ethanol yields and reduced the level of exogenous enzyme loadings required (Zhang et al., 2011). Another study showed that the expression of cellobiohydrolases in transgenic corn also reduced the saccharification cost associated with the production of fermentable sugars (Harrison et al., 2014).

To increase the accessibility of cellulose for fermentation, positive progress has also been made through decreasing the lignin and non-cellulosic polysaccharide content in biomass, which has led to improved saccharification efficiency in a number of crop species (Harris et al., 2013; Loqué et al., 2015). Successful examples in non-food, lignocellulosic feedstock with down-regulated cell wall biosynthetic pathways and reduced recalcitrance include switchgrass (Fu et al., 2011; Shen et al., 2013; Baxter et al., 2014) and poplar (Biswal et al., 2015; Bryan et al., 2016). Promising results in the engineering of low-lignin switchgrass have been reported; the reduction of lignin content of this material is stable in the field and is achieved without penalty with respect to disease susceptibility (Baxter et al., 2014). A non-GM approach to the identification of poplar lines with relatively low lignin contents has been achieved by analysis of natural variation and the exploitation of genetic variation therefore provides opportunities for ameliorating the recalcitrance problem (Bhagia et al., 2016). During anaerobic fermentation, other wall components can also inhibit the conversion process. For example, acetate substituents on pectic polysaccharides, non-cellulosic polysaccharides and lignin can inhibit enzymatic hydrolysis by blocking cleavage sites for endo-lytic enzymes (Gille and Pauly, 2012). Furthermore, un-dissociated acetic acid can be toxic to microorganisms at high concentrations and this inhibits cellular growth rates. Some fermenting organisms such as *Saccharomyces cerevisiae* tolerate concentrations up to 4 g/L with no negative effect on ethanol yields if the substrate is glucose. However, if the substrate is xylose, inhibition occurs at a much lower concentration (1.5 g/L), which reduces cellular growth by 15% and consequently decreases ethanol yields by 50% (Helle et al., 2003). If thermochemical conversion processes are adopted for the production of hydrocarbon based biofuel over conventional anaerobic fermentation, the complexity and composition of biomass polymers are less of a concern.

Harris et al. (2013) suggested that increasing the amount of cellulose might also increase the amount of biomass and energy density of feedstocks, particularly where thermal combustion methods are used to convert biomass directly to liquid biofuels. In contrast to the successful outcomes obtained by tailoring biofuels crops to generate less lignin, success in manipulating genes to increase cellulose content is limited to manipulating genes that indirectly affect cellulose production, such as carbon partitioning (Coleman et al., 2009), hydrolysis of cellulose (Park et al., 2004; Shani et al., 2004) or a reduction in lignin content that was compensated for by an increase in cellulose (Hu et al., 1999; Li et al., 2003; Leplé et al., 2007). Direct manipulation of cellulose synthase subunits has proved relatively unsuccessful. Attempts to increase cellulose content in woody poplar (Joshi et al., 2011) have shown that over-expressing a single poplar secondary cell wall (SCW) *PtdCesA8* gene driven by the CaMV 35S promoter resulted in transgenic plants with dwarfism, shoot tip necrosis, transcript suppression, a reduction in cellulose and collapsed xylem morphology. These phenotypes resembled those observed in transgenic barley *35S:HvCesA4* (Tan et al., 2015). Despite the systematic attempt to up-regulate individual *HvCesA* genes involved in both primary and secondary cell wall biosynthesis, Tan et al. (2015) were unable to generate increased cellulose contents in the transgenic lines. Instead, attempts to perturb *HvCesA* gene expression generally causes gene silencing and extreme phenotypes. The results from both poplar and barley suggest that a relatively narrow range of cellulose levels is maintained by tight regulatory mechanisms in higher plants (Tan et al., 2015). The results further showed that over-expression of a single *HvCesA* gene driven by the CaMV 35S promoter was not enough to increase cellulose content in the cell wall of barley. When native barley and maize *CesA* promoters were used to drive the *CesA* genes, aberrant phenotypes and silencing effects were ameliorated but no significant increases in cellulose were observed (Tan, 2013). Additionally, increasing transcript levels of all secondary cell wall *CesA* genes in *Arabidopsis* by over-expressing a transcription factor did not translate into an increase in cellulose, again suggesting tight regulatory mechanisms (Tan, 2013).

Despite the wide species variation between poplar and barley, manipulating cellulose content proved to be very difficult in both species and this effect is likely to be observed in other species too. We have concluded that the central importance of cellulose content in plant structure means that plants have evolved mechanisms to resist any major upward or downward movement of cellulose content.

## Challenges In Bioethanol Production – From Pre-Treatment To Fermentation

### Pre-treatments

**FIGURE 2:**
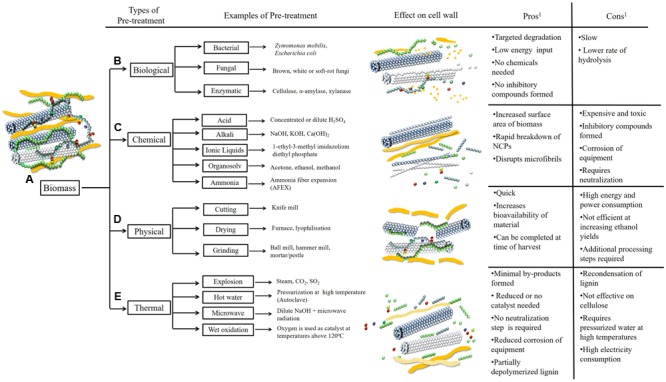
**Selected pre-treatment methods differentially affect the breakdown and liberation of polymers from the cell wall.** Lignocellulosic biomass is a complex network of carbohydrate and non-carbohydrate polymers **(A)**. In its native form plant biomass is usually recalcitrant to conversion and fermentation processes. Pre-treatment is the initial processing step used to convert raw biomass into a form that can be more readily hydrolyzed. Biological pre-treatments are efficient at removing lignin from the network, leaving a carbohydrate-enriched fraction **(B)**. Chemical pre-treatments may result in complete breakdown and fragmentation of cell wall components **(C)**. Physical pre-treatments are used to reduce the particle size of the biomass **(D)**. The use of thermal pre-treatments may loosen bonds between and within polymers but lignin is not completely removed **(E)**^1^ (Mosier et al., 2005; Kumar et al., 2009; Takara and Khanal, 2012).

Biomass pre-treatment refers to the initial steps taken to convert biomass from its native recalcitrant state into a form that can be more readily hydrolyzed (Mosier et al., 2005). Methods used to facilitate the depolymerization of polysaccharides into monosaccharides or oligosaccharides include the use of physical pre-treatments that reduce the particle size or moisture content of the biomass, chemical treatments, thermal exposure or biological treatments (**Figure 2**) (Agbor et al., 2011) in a feedstock-dependent manner. The composition of the biomass and the severity of the pre-treatment directly affect the degradation of polymers, the percentage of residual carbohydrates, cost and the efficiency of the subsequent hydrolysis. For example, if it is necessary to remove lignin from the cell wall matrix, biological pre-treatments using white-rot fungi have been shown to be effective (Kumar et al., 2009). Chemical pre-treatments with alkali, acid and other solvents are effective for subsequent hydrolysis of non-cellulosic polysaccharides and for disrupting cellulosic microfibrils, whilst thermal pre-treatments partially depolymerize lignin, although re-condensation may occur (Kumar et al., 2009).

The use of pre-treatments is often considered to be essential in the processing of lignocellulosic biomass into a more amenable form. However, pre-treatments can be costly in terms of time and energy input and may necessitate additional downstream processing to remove acidic or alkaline residues. For example, there are a number of compounds that can accumulate during pre-treatment that lower the efficiency of carbohydrate conversion to ethanol, some of which interact antagonistically. These inhibitory compounds have been categorized into three major groups: weak acids, furan derivatives, and phenolic compounds (Palmqvist and Hahn-Hägerdal, 2000), in which weak acids have been identified as the main inhibitor to yeast (Huang et al., 2011). The accumulation of inhibitory compounds causes an extension of the lag phase (metabolic preparation for growth and division), slower growth rates, lower cell density, and decreased ethanol conversion during fermentation (Palmqvist and Hahn-Hägerdal, 2000; Heer and Sauer, 2008). One of the key microbial inhibitors present in hydrolysates, furfural, extends the lag phase and negatively affects the growth and carbon metabolism of fermenting microorganisms (Heer and Sauer, 2008). Some yeast strains have a detoxification mechanism to circumvent the accumulation of furfural, converting it to furfural alcohol (Palmqvist and Hahn-Hägerdal, 2000). Many of the 60 organic acids commonly found in lignocellulosic hydrolysates have also been shown to reduce yeast growth and ethanol yield by inhibiting monosaccharide metabolism and causing intracellular anion accumulation (Almeida et al., 2007; Heer and Sauer, 2008; Huang et al., 2011).

The type and amount of inhibitory compounds produced during pre-treatment are dependent on the composition of the lignocellulosic biomass and the conditions under which the raw material is treated. However, conditioning the substrate prior to introducing the fermenting organism may minimize the inhibitory effects of these compounds. For example, concentrations of formic acid above 4 g/L inhibit yeast such as *Saccharomyces cerevisiae* but the inhibitory effects can be overcome by buffering the pH between 6.0 and 9.0 (Huang et al., 2011). Improved biotechnological strategies and pre-treatment conditions may reduce the need to employ biological detoxification or potentially costly methodologies to remove inhibitory compounds; such options have been investigated in detail elsewhere (Nichols et al., 2005, 2010; Mohagheghi et al., 2006; Qing et al., 2010; Parawira and Tekere, 2011). Alternatively, different downstream processing strategies such as fast-pyrolysis, slow-pyrolysis, esterification, *trans*-esterification, and hydrothermal liquefaction may be used, because inhibitory compounds generated during pre-treatment have no effect on the production of biofuel using these technologies (Atabani et al., 2012; Borges and Diaz, 2012; Isahak et al., 2012; Xiu and Shahbazi, 2012).

A number of studies have been focused on tailoring pre-treatments to specific feedstocks (Torres et al., 2013; Rarbach et al., 2014), to achieve optimal results (Galbe and Zacchi, 2007) and to minimize the production of inhibitory compounds (Cho et al., 2009; Liu and Blaschek, 2010; Jönsson et al., 2013). As shown in surface response methodology studies, even moderate changes to parameters such as pH, time, solvent and temperature can change the yield, purity, structure and size of polysaccharides extracted (Lorbeer et al., 2015) and the efficiency of enzymatic hydrolysis following pre-treatment (Ferreira et al., 2009; Qi et al., 2009). Unfortunately, few studies have taken into consideration the economic cost of each modification if it were to be applied at an industrial scale. Although informative, this tailoring methodology is generally specific for a single or a narrow range of feedstocks.

The criteria by which a pre-treatment method is commonly assessed (Galbe and Zacchi, 2007) can be summarized as follows:

(i) The pre-treatment should result in high recovery of monosaccharides.
(ii) It should result in complex polysaccharides such as cellulose being readily digested if subsequent enzymatic hydrolysis is required.
(iii) There should be minimal production of inhibitory compounds and hazardous or toxic waste generated.
(iv) Detoxification is not required and the hydrolysate can be directly fermented.
(v) The capacity for value adding, making use of co-products or generation of energy (i.e., electricity) should be maintained.
(vi) The pre-treatment should have low capital and operational costs.

#### Biological Pre-treatments

Genome sequencing of the lignocellulose-degrading “white rot” fungi (Floudas et al., 2012) and the termite gut microbiome community (Warnecke et al., 2007) has resulted in dramatic advances in enzyme discovery and biomass processing. Biological pre-treatments are reliant on the use of microorganisms or more specifically on the enzymes secreted by these organisms. Biological pre-treatments predominantly employ fungi (brown, white, or soft-rot), which target lignin and cellulose and, to a lesser degree, non-cellulosic polysaccharides. White rot-fungi represent one of the most effective biological treatments for lignin degradation because this fungus produces lignin degrading enzymes (laccases and peroxidases) that are regulated by carbon and nitrogen sources (Kumar et al., 2009). For example, the white-rot fungus *Pleurotus ostreatus* has been shown to remove 41% of the acid insoluble lignin in rice straw after 60 days of exposure, leaving a carbohydrate enriched fraction (Taniguchi et al., 2005). The use of fungi in conjunction with other pre-treatments, such as bio-organo-solvation, has been shown to increase ethanol yields by 1.6 times compared with non-fungal treated biomass and, concurrently, reduced electricity consumption for ethanolysis by 15% (Itoh et al., 2003). Bacterial strains in combination or as single pre-treatments have likewise been investigated (Kurakake et al., 2007). Microbial pre-treatments are advantageous because they facilitate downstream processing of biomass, require low energy inputs and circumvent the need for high temperatures or the use of chemicals. However, the rate of treatment is slower (5-8 weeks) and results in a lower rate of hydrolysis compared with other pre-treatments (Hatakka, 1983).

#### Chemical Pre-treatments

Acidic solutions have been shown to be effective pre-treatments that partially hydrolyze non-cellulosic polysaccharides (mainly xylan) and disrupt cellulose microfibrils (Kumar et al., 2009; Takara and Khanal, 2012). These modifications often increase the surface area available for enzyme attack by swelling the biomass material. Dilute sulfuric acid pre-treatments are considered a more economically viable option for improving the conversion of lignocellulosic biomass to ethanol, but they are still costly when modeled against a scenario reflective of an ideal pre-treatment (Eggeman and Elander, 2005). Dilute acid pre-treatments are performed at high temperatures (180°C) for short periods of time or at lower temperatures (about 120°C) for longer times (30-90 min) (Alvira et al., 2010). Hydrochloric acid, phosphoric acid and nitric acid are also commonly used, although with lower hydrolysis yields than sulfuric acid (Israilides et al., 1978; Goldstein and Easter, 1992; Kim et al., 2000; Saha et al., 2005). The use of concentrated acids is considered an unfavorable approach, because the chemicals produce higher yields of inhibitory compounds, can corrode equipment more rapidly, and require subsequent neutralization steps. For acid pre-treatments, additional costs might be incurred for the neutralization and disposal of the acid or salts following the pre-treatment steps.

Likewise, chemical pre-treatment studies have included the use of alkaline reagents, most commonly sodium hydroxide or calcium hydroxide. Studies show alkaline methods are unfavorable as the reagent may be converted to salt during the reaction, which is costly or impossible to remove (Mosier et al., 2005). Ammonia (ammonia fiber expansion, AFEX) has also been considered as a chemical biomass pre-treatment (Harmsen et al., 2010; Maurya et al., 2015). More recently, non-volatile solvents classified as ionic liquids (IL) have been used for the dissolution of the plant cell wall and regeneration of polysaccharides such as cellulose (Mäki-Arvela et al., 2010). The properties of ionic liquids such as quaternary ammonium ILs, *N*-alkyl-pyridinium ILs, *N*-alkyl-isoquinolinium ILs, and 1-alkyl-3-methyl-imidazolium ILs can be tuned by appropriate selection of anions and cations (Liu et al., 2012). Ionic liquids have the added advantage of being recoverable and reusable, exhibit high thermal stabilities and negligible vapor pressure. However, ionic liquids tend to be viscous, which reduces mass transfer and increases the energy requirements for substrate mixing (Holm and Lassi, 2011). The efficiency of different ionic liquid solvents for the degradation of cell wall polysaccharides has been compared in detail elsewhere (Mäki-Arvela et al., 2010).

#### Physical Pre-treatments

Physical techniques that are used to enhance the digestibility of biomass usually involve size reduction by chipping, shredding, grinding or milling and generally increase the surface area available for subsequent enzymatic degradation. Physically altering the plant biomass and hence the potential yield of biofuel per gram of biomass, begins at harvest. The types of physical treatments, as well as the nature of the biomass, change the available surface area of the harvested material, together with the degree of polymerization of polysaccharides, the moisture content and the degree of cellulose crystallinity (Agbor et al., 2011). However, this process can be unfavorable with respect to the mass-energy balance of the entire process, because of the high energy input required. For example, switchgrass physically pre-treated using a hammer mill required an energy input of 27.6 kW h t^-1^ to reduce particle size to a 3.2 mm screen size (Mani et al., 2004). It is generally accepted that the effect of particle size on polysaccharide degradation is related to the surface area available to enzymes, in which there is an inverse relationship between the rate of conversion and particle size (Al-Rabadi et al., 2009). Other studies have suggested that although particle size is an indicator of surface area, its correlation to the total accessible volume is much less predictable (Vidal et al., 2011). Similarly, there appears to be no consensus on how the moisture content of biomass impacts upon the rate of polysaccharide degradation and therefore may be feedstock dependent. For example, some studies suggest that increased moisture content reduces the relative severity of pre-treatments, generating improved solids and non-cellulosic-derived carbohydrate recovery (Cullis et al., 2004). Other studies show that increasing the moisture content of corn fiber (from 30 to 150% dry weight basis) has no significant effect on the rate of enzymatic hydrolysis following AFEX pre-treatment (Moniruzzaman et al., 1997). In another study it was found that by increasing the moisture content of bagasse from 12 to 80% the permeability of the biomass to SO_2_ was enhanced, subsequently generating higher yields of ethanol (Ewanick and Bura, 2011).

#### Thermal Pre-treatments

Hot, pressurized water at 120-480 psi and at temperatures above 120°C, more effectively penetrates biomass and increases the susceptibility of the cell wall to hydrolysis of its non-cellulosic polysaccharides and amorphous cellulose constituents (Bobleter, 1994). Examples of thermal pre-treatments include explosion (CO_2_ or SO_2_), hot water (autoclave), microwave (microwave radiation with NaOH), oxidative (H_2_O_2_) and wet oxidation (Harmsen et al., 2010; Maurya et al., 2015). During thermal pre-treatment, lignin is partially depolymerized and solubilized. The complete delignification of the cell wall is not possible using this method alone, due to the re-condensation of soluble components released from lignin (Alvira et al., 2010). In general, liquid hot water pre-treatments are attractive from a cost-saving perspective because no catalyst is needed and there is minimal corrosion of equipment, compared with acid treatments (Mosier et al., 2005), but there is a trade off in the breakdown of cell wall polysaccharides. To increase the severity of the pre-treatment, combining thermal pre-treatments with alkaline hydrolysis was shown to increase the rate of lignin oxidation, the rate of enzymatic saccharification and, additionally, no microbial inhibitory compounds such as furfural were formed (Bjerre et al., 1996). Another advantage of thermal pre-treatments is that the costs associated with particle size reduction are fully exploited as smaller biomass is more rapidly broken down in hot water. Further advantages of using thermal rather than chemical pre-treatments include lower concentrations of solubilized non-cellulosic polysaccharides and lignin products, lower chemical usage, and no requirement for neutralization of the resultant hydrolysate (Alvira et al., 2010).

#### Enzymatic Pre-treatment

**FIGURE 3:**
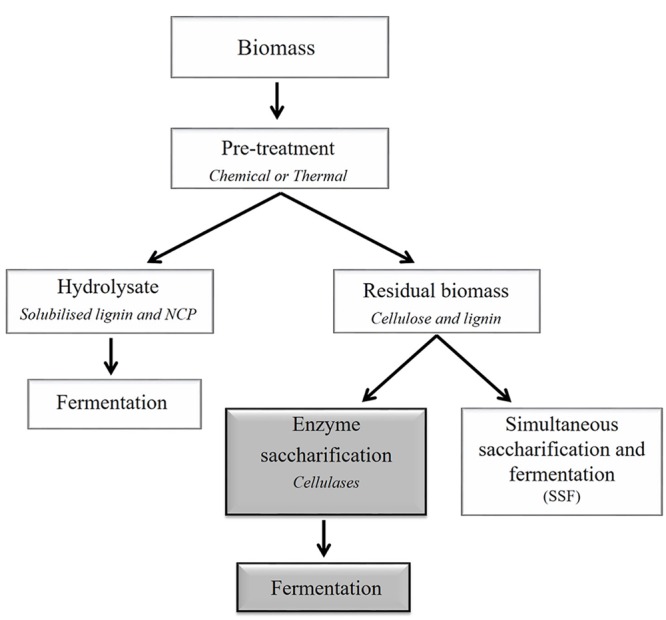
**Simplified outline of a generalized processing scheme for the conversion of plant lignocellulosic biomass to bioethanol.** Compositional data are informative when selecting appropriate downstream processing methods, including pre-treatments. Pre-treatments are used to break or loosen the cell wall matrix. For example, chemical and thermal pre-treatments separate the biomass into two fractions, namely the hydrolysate and the insoluble residual biomass. The hydrolysate is enriched in solubilized carbohydrates that are predominantly derived from non-cellulosic cell wall polysaccharides (NCPs) and may be fermented to ethanol. The residual biomass is predominantly cellulose and lignin, which can be further processed using enzymes and fermented. This two-step process (separate hydrolysis and fermentation, SHF) is represented by dark gray boxes). Alternatively, the residual biomass may be converted to ethanol in one step via simultaneous saccharification and fermentation (SSF). It should be noted that the figure does not depict a method that would simultaneously deconstruct (solubilize) the carbohydrates and ferment the sugars, such as consolidated bioprocessing (Olson et al., 2012).

Depending on the efficiency of the pre-treatment, enzymatic hydrolysis can be used to completely depolymerize polysaccharides to their respective monosaccharide constituents (**Figure 3**). In the biofuels context, hydrolysis refers to the catalytic decomposition of polysaccharides into fermentable sugars through the action of specific enzymes. The efficiency of action of these enzymes is impacted by their substrate specificities, in addition to their kinetic properties and to enzyme concentration. The substrate properties include the degree of polymerization, relative crystallinity, accessible surface area or bonds, and the presence of lignin. These parameters differ between feedstocks (Zhang and Lynd, 2004). The three types of cellulase enzymes commonly used for lignocellulosic biomass processing are endo-(1,4)-β-glucanases, exo-β-glucanases, and β-glucosidases. Endoglucanases increase the number of chain ends and significantly decrease the degree of polymerization by hydrolyzing the interior glucosidic linkages of cellulose molecules (Zhang and Lynd, 2004). Exoglucanases shorten the chains incrementally, usually by hydrolyzing glycosidic linkages at the non-reducing ends of cellulosic chains or released oligosaccharides (Kumar et al., 2008). The β-glucosidase enzymes act on short oligomers such as cellobiose, which is a disaccharide produced from partial hydrolysis of cellulose by the exoglucanase, cellobiohydrolase. If biological or thermal pre-treatments are used, the biomass may also need to be treated with enzymes specific to non-cellulosic polysaccharides to liberate additional pentose and hexose monosaccharides. For xylose-based non-cellulosic polysaccharides, xylanases may be used to degrade linear (1,4)-β-xylan chains. Other enzymes that may be used to hydrolyse non-cellulosic polysaccharides include mannanases, galactosidases, galactanases, arabinanases, and a range of pectin-degrading enzymes (Dhawan and Kaur, 2007; Menon and Rao, 2012). Feedstocks that have less recalcitrant cell walls, due to lower lignin levels or to lower cellulose crystallinity, may have similar hydrolysis rates for treated and non-treated material. Although the use of enzymes may increase the amount of available sugar for fermenting organisms, it may become a limiting factor when converting from small scale to large scale production.

### Fermentation

*Saccharomyces cerevisiae* is one of the most common yeasts used for the production of ethanol biofuel (Hahn-Hägerdal et al., 2006), although the efficiency of other fermenting organisms such as *Pichia stipitis, Kluyveromyces* sp., and *Hansenula polymorpha* have been investigated (Weber et al., 2010). Bacteria may likewise be an option for fermentation, as many species can utilize both hexose (C6) and pentose (C5) sugars although issues with catabolite repression have been reported (Hahn-Hägerdal et al., 2006). To overcome these limitations, bacteria may be metabolically engineered to generate recombinant strains capable of producing ethanol from plant biomass. One of the pioneering studies in this area engineered a recombinant strain of *Escherichia coli* containing plasmid-borne genes (pyruvate decarboxylase and alcohol dehydrogenase) from *Zymomonas mobilis* which encode enzymes for the ethanol pathway (Alterthum and Ingram, 1989).

Alcoholic fermentations involve complex biochemical and enzymatic reactions that occur at reduced oxygen concentrations and which slow down as conversion nears completion or as ethanol concentrations build to inhibitory levels. Fermentations can also slow down or stop in response to environmental stresses such as extremes of pH, osmolarity, temperature, oxygen levels, decreased sugar utilization by the yeast, decrease in cell growth, or intolerance to reactants and/or end products (Salmon, 1996; Bisson, 1999). The fermenting competence of the microorganism and its dependence on the sugar composition of the sample will also influence the yield and rate of conversion (Liccioli, 2010). For lignocellulosic ethanol to be commercially viable on a large-scale, the fermenting organism will need to tolerate a range of environmental conditions and stresses, and must be capable of metabolizing the specific sugars present in the raw material. Fermentations in which the predominant carbohydrate source comprises simple sugars can be converted directly to ethanol. If the solution has higher molecular weight polymers or a high concentration of unusual sugars, additional enzymes might be needed. The addition of enzymes to a fermentation is known as a simultaneous saccharification and fermentation, SSF. One limitation for this processing method is that commercial cellulases (∼50°C) and yeast (<35°C) have different optimal temperatures. For both biological processes to occur concurrently without additional operational costs thermophilic organisms such as *Clostridium* sp. may be used (Weber et al., 2010).

The activation of the fermentation pathway begins when sugar molecules are transported across the plasma membrane. The transporters have a higher affinity for certain sugars in a heterogeneous sugar solution, but affinity decreases with the depletion of the preferred sugar(s) over time (Berthels et al., 2004; Perez et al., 2005). Upon entry into the yeast cell, the sugar molecules are partially oxidized often through the glycolytic pathway and some adenosine-5′-triphosphate (ATP) is generated. Both glucose and fructose are phosphorylated by hexokinase enzymes into fructose-6-phosphate, but at differing rates and with a preference for glucose (Jang et al., 1997). The final product of glycolysis, pyruvate, will be subject to alcoholic fermentation in anaerobic conditions and the concomitant regeneration of NAD from NADH enables the glycolytic pathway to continue. However, not all sugars in heterogeneous substrates from plant cell walls can be converted to ethanol via the metabolic pathways that operate in yeasts. The accumulation of sugars that cannot be immediately metabolized results in arrested fermentation. Ethanol itself can also inhibit the yeast by increasing plasma membrane disruption, disrupting passive proton flux, damaging intracellular enzymes and causing cell death (Bisson and Block, 2002; Stanley et al., 2010). Inhibitory compounds such as furfural, produced as a by-product from certain pre-treatments, directly inhibit glycolytic enzymes and aldehyde dehydrogenase activity. This leads to the accumulation of acetaldehyde, which lengthens the lag phase for microorganisms such as *S. cerevisiae* and *E. coli* (Palmqvist et al., 1999; Saha and Cotta, 2011).

As a result of these effects, genetic manipulation and optimization of fermenting microorganisms and methodologies to produce optimal yields of ethanol from lignocellulosic biomass are areas of much interest. For example, *S. cerevisiae*, cannot normally ferment pentose sugars, rendering up to 45% of the sugars in some raw feedstocks unusable for ethanol production (Kumar et al., 2009). To circumvent this limitation, *S. cerevisiae* can be modified to improve ethanol production capability through a variety of techniques including both recombinant and non-recombinant methodologies. In grasses for example, pentose rich heteroxylans are major constituents of cell walls and hence of crop residues or specialist grasses used for biofuel production. For many years studies have been targeted to improving the rate at which pentose sugars are transported and metabolized by yeast cells. Recombinant DNA approaches have introduced genes into yeast from organisms capable of naturally fermenting pentose sugars, such as fungi, insects, ruminant guts and *P. stipitis* (Hahn-Hägerdal et al., 2007). In addition, genetic manipulations of yeast in conjunction with the expression of exogenous genes in a heterologous system in microorganisms for the production and regulation of saccharolytic enzymes such as cellulases can result in the breakdown of intractable polysaccharides and their conversion to ethanol to occur in a one-step process known as consolidated bioprocessing (CBP) (Lynd et al., 2002). However, some organisms have the genetic machinery to synthesis enzymes naturally, which could be further exploited. For example, *Kluyveromyces marxianus* can hydrolyse fructans and convert the released monomers to ethanol, by secreting fructanases (Arrizon et al., 2011). These types of approaches enable a ‘natural’ simultaneous saccharification and fermentation process that eliminates the costs associated with exogenous enzyme addition.

Another non-recombinant approach that has been applied to wine making (McBryde et al., 2006) and more recently to biofuel production is the use of adaptive evolution. Adaptive evolution is defined as the occurrence of advantageous mutations in a population as a response to specific challenges (Foster, 1999). Adaptive mutations can arise spontaneously from exposure to a particular carbon source or as a physiological response to a particular stress (Cairns et al., 1988). Thus, yeasts can be selectively pressured into adapting to specific environmental conditions or they can be conditioned to have a preference to efficiently utilize either a specific carbon source or a range of carbohydrates (Liccioli, 2010). Adaptive evolution can be achieved by using a batch culture approach, in which a percentage of the population is isolated and transferred to fresh media at intervals. It can also be conducted by maintaining the culture in an isolated environment and periodically replacing exhausted media with fresh media (Liccioli, 2010). In Parreiras et al. (2014), adaptive evolution was used to generate a *S. cerevisiae* strain that could ferment xylose from AFEX pre-treated corn stover hydrolysate under anaerobic conditions. This enhancement in pentose fermentation efficiency was attributed to a missense mutation acquired during evolution which reduced the formation of the xylose isomerase inhibitor, xylitol. As shown in this study, non-recombinant modifications such as adaptive evolution can be a promising approach to identify superior organisms that utilize all carbohydrates in a plant material which ultimately increases the biofuel yields achieved.

Advances in genomic studies of both feedstocks and microorganisms have presented new opportunities for developing more efficient fermentation-based conversion systems (Rubin, 2008). Plant biomass contains a broad range of associated microorganisms but the study of these populations is hampered by the fact that less than 1% of microorganisms present in many natural environments can be cultured *in vitro* (Aden and Foust, 2009). Emerging methodologies, such as Next-Generation Sequencing (NGS) of microbial populations, are overcoming these problems. Comparative metagenomic and functional analysis of the digestive tract microbial populations collected from lignocellulose-degrading hosts such as termites and ruminants have increased our understanding of cell wall deconstruction and helped to identify a diverse set of bacterial genes for cellulose and xylan hydrolysis (Warnecke et al., 2007; Brulc et al., 2009). It has been proposed that microorganisms inherent to bioenergy feedstocks may provide the best enzymes for deconstructing plant cell walls for biofuel production. A recent study demonstrated that fungi isolated from decaying leaves of energy grasses were superior to the commercial bioconversion fungus *Trichoderma reesei* when applied to *Miscanthus* biomass (Shrestha et al., 2015). Thus, mining the natural microflora of lignocellulosic feedstocks could result in the isolation of microbes that outperform commercially available strains, where directly fermenting the biomass in its native form could obviate the need for inoculation.

## Integration Of Multiple Technologies For Lignocellulose Conversion

Many of the challenges associated with the cost-efficient conversion of lignocellulosic plant cell wall residues to ethanol have been tackled through an integrated approach to obtaining a positive mass-energy balance by co-locating companies or facilities that allow the exchange of energy and product streams (Chum et al., 2015). Co-locating lignocellulosic biomass to ethanol conversion facilities with traditional power plants enables waste steam from the power plant to be used for pre-treatment of biomass sources such as wheat straw (Chum et al., 2015). Following the steam pre-treatment, ethanol can be produced from the biomass source through simultaneous enzymatic hydrolysis and fermentation and the residual lignin fraction can be pelletized and returned for combustion in the power plant. The five-carbon molasses material, which originates from the high concentration of heteroxylans in grass or cereal crop residues, can be used for cattle feed or can be further fermented by anaerobic microorganisms capable of metabolizing pentose to ethanol (Janssen et al., 2013). Alternatively, gasifiers might be used for conversion of biomass or biomass products to high quality gases for co-firing with the power plant’s combustor (Chum et al., 2015).

Although these integrated facilities have shown great promise at the ‘demonstration plant’ level (Chum et al., 2015), the construction and commissioning of full scale facilities have been delayed by falling oil prices and other uncertainties (Reboredo et al., 2016). For example, the operation of a demonstration plant in Kalundborg, Denmark, as described in Chum et al. (2015) was put on standby in 2015 (McMillan et al., 2015). Other commercial scale plants and planned commercial plants have likewise been closed or construction has been postponed (International Energy Agency, 2016). The operational status of various biofuel facilities around the world has been compiled and updated by the International Energy Agency (http://demoplants.bioenergy2020.eu/).

Other questions are also raised in consideration of large bioethanol production facilities that use plant cell wall material as a source of biomass. If a facility is constructed to use a single biomass source, to what extent does the process need to be tuned for seasonal variations in that biomass or if the company wishes to use alternate or multiple biomass sources. Is bioethanol production ever going to be highly profitable, given the challenges discussed in the sections above? Should we focus on manipulating the biomass source, the various pre-treatments and their more specific conditions, the fermenting microorganisms, added enzymes, or all of these in parallel? In considering these challenges, it becomes apparent that an efficient, ‘universal’ conversion technology, through which multiple feedstocks could be used with minimal changes in the conversion process, would provide an enormous boost for renewable biofuel industries that rely on plant residues as biomass sources.

## Cellulosic Biofuel Conversion Methods And The Prospect Of Emerging Technologies

Several new technologies to harness the energy content of biomass and make it more available for a variety of uses are now emerging. Of these, thermochemical processes look particularly promising to overcome the existing problems related to biochemical conversion, such as prolonged reaction times, low conversion efficiency by enzymes and microorganisms, and high production costs (Sims et al., 2010; Raheem et al., 2015). Thermochemical processes allow biomass to be transformed directly into liquid fuels, thus increasing the efficiency of the process and hence the energy density of the product and will often enable easier handling, distribution and storage of the biofuel product, using existing infrastructure. The capacity for such bio-oils to be incorporated into existing infrastructure established for petroleum-based fuel has resulted in the term ‘drop-in’ biofuels. Three thermochemical conversion methodologies are routinely used according to the oxygen content in the process: combustion (complete oxidation), gasification (partial oxidation) and pyrolysis (thermal degradation without oxygen) (Silva et al., 2015).

The production of crude-like oil, or bio-oil, from thermal decomposition technologies such as pyrolysis and hydrothermal liquefaction may eliminate the necessity to fractionate plant biomass as these methods are designed to be ‘feedstock agnostic’, insofar as they are amenable to the use of multiple feedstocks or fractions with highly variable compositions. Biomass pyrolysis occurs at temperatures ranging from 200 to 750°C in the absence of oxygen and generates three main products: renewable bio-oil, char, and gases (Raheem et al., 2015). Slow pyrolysis is predominantly employed for producing charcoal while fast pyrolysis is used to increase bio-oil yields (Bridgwater, 1999, 2010; Bridgwater et al., 1999; Bridgwater and Peacocke, 2000). The latter can be used directly in a generator to produce electricity or further refined as a transportation fuel. However, the application of the technology has been limited by its requirement for low moisture biomass and by difficulties experienced in overcoming issues related to the high reactivity, high acidity and high oxygen contents of biomass-derived pyrolysis oils. Thus, biomass-derived fast pyrolysis oils contain water that cannot be readily separated, and this limits their gross calorific values to around 17 MJ/kg (Bridgwater, 2010). Venderbosch and Prins (2010) have reviewed the principles of fast pyrolysis and the key technologies, including fluid beds, rotating cone and vacuum pyrolysis, ablative and twin screw pyrolysis.

Re-emerging technologies such as hydrothermal liquefaction (HTL), which are more robust and can accommodate multiple biomass sources, have a major advantage over methods such as pyrolysis in the fact that they are able to utilize wet biomass without the need for costly drying steps (Akhtar and Amin, 2011). Hydrothermal liquefaction is a thermochemical process that uses pressures in the range 150-180 bar and temperatures in the range 300 to 350°C (Goudriaan and Peferoen, 1990; Behrendt et al., 2008; Toor et al., 2011), where water approaches its critical point and becomes a highly reactive medium that penetrates solid biomass and mimics geological processes that generated our current reserves of fossil fuels. Pre-treatments are not required. During the HTL process, many of the small compounds released are unstable and reactive, forming larger hydrocarbons. CO_2_ is also ultimately converted from the oxygen in the biomass which is removed by decarboxylation and dehydration (Goudriaan and Peferoen, 1990; Toor et al., 2011). Release of the pressure after the reaction is complete leads to phase separation and the relatively easy separation of the product, which is often called bio-oil or bio-crude. The bio-crude oil has a relatively high energy density of 33.8-36.9 MJ/kg and lower oxygen content (5-20%) compared with pyrolytic bio-oil, thus allowing the possibility of blending with traditional hydrocarbon fuels without emulsification. By way of comparison with the energy density values cited above for HTL, the energy density of bioethanol is estimated at 26.4 MJ/kg (Fersi et al., 2012) while current fossil fuel (petrol) is estimated at 44.4 MJ/kg (Ayalur Chattanathan et al., 2012). The chemical properties of bio-oil are highly dependent on the biomass composition as each component (i.e., polysaccharides, lignin, protein and lipid) produces a distinctive spectrum. Lignin remains in the residue fraction and non-cellulosic polysaccharides are readily reduced to saturated hydrocarbons (Akhtar and Amin, 2011).

Due to the requirement for high pressures, HTL processes usually require more expensive reactors than pyrolysis processes. However, the reactor is the sole component of the process, and overall the costs of hydrothermal liquefaction and fast pyrolysis may be similar (Nabi et al., 2015). An important technological development will be the design of continuous flow systems that obviate the repeated need for batch wise temperature application. Elliott et al. (2015) have reviewed a continuous-flow processing system for microalgae and lignocellulosic feedstocks, together with the downstream processing of HTL products. Energy balances and conceptual process costs have been calculated and the commercial potential of this technology has been assessed (Elliott et al., 2015). In related work, Raheem et al. (2015) have reviewed the literature on thermochemical processing of microalgal biomass sources and noted that microalgal pyrolysis oils are more stable and less oxygenated than pyrolysis oils from lignocellulosic biomass and re-affirmed earlier suggestions that thermal liquefaction of microalgae is a very promising pathway to higher quality bio-oils, with calorific values close to those of petroleum oil. New technologies such as continuous flow vortex fluidic production have been used in the direct conversion of sunflower oil to high purity biodiesel without the need for saponification, co-solvents or complex catalysts (Britton and Raston, 2014).

Nevertheless, challenges remain before bio-oils can be considered as commercially viable ‘drop-in’ fuels. Both HTL and pyrolysis oils can suffer from high acidity and high iodine levels, which require changes to current storage and transfer facilities. Efforts to upgrade HTL bio-oil to fossil fuel specifications are underway via solvent hydrogenation, catalytic cracking, esterification, and hybrid processes (Ramirez et al., 2015). Concern may arise from the release of by-product from the thermochemical conversion into the environment. Technology such as catalytic hydrothermal gasification (CHG) has been in development to clean up organic waste found as aqueous byproduct in HTL (Elliott et al., 2013, 2015). The gas can be turned into energy (e.g., heat/electricity) and consists mainly of carbon dioxide and methane with minimal amounts of pollutants such as carbon monoxide or hydrocarbon (Elliott, 2008).

Furthermore, the HTL technology has been applied to a wide range of biomass and organic waste materials, including woody biomass (Zhu et al., 2014), mixed culture algae (Elliott et al., 2013; Chen et al., 2014b; Ou et al., 2015), manure (Chen et al., 2014a), food and agricultural waste (Pavlovič et al., 2013) and municipal waste (Minowa et al., 1995). It has been demonstrated that it is possible to use components of renewable bio-crude from HTL of lignocellulosic biomass, unrefined except for distillation, as a fuel for modern diesel engines when blended with automotive diesel fuel (Nabi et al., 2015). In spite of its potential for hydrothermal production of renewable fuels, research in this area is still in its infancy and the technology has not yet achieved commercial scale.

## The Importance of Government Policies for a Successful Biofuels Industry

The development of viable renewable transport fuel industries has relied heavily on positive action and financial support from central governments. In this context, positive measures put in place for cellulosic biofuels are mandated and enforced in countries such as the USA and Europe. The US renewable Fuel Standard (RFS) program requires renewable fuel to be blended into transportation fuel in increasing amounts each year, reaching 136 billion liters by 2020. In 2013, US fuel ethanol production reached 54 billion liters. In April 2015, via the ‘indirect and use change (iLUC) Directive’, the European Parliament capped the production of biofuel crops grown on agricultural land to 7% and increased its emphasis on advanced biofuel production to meet its 2020 target of achieving 10% renewables in transport fuels. Thus, Europe has indicated its determination to ensure that agricultural land is not used for specialist biofuel crops.

By way of contrast to Europe and the USA, the Australian government has been relatively slow to provide the regulatory environment in which a viable biofuels industry will thrive. While the transport sector accounts for 40% of the country’s energy consumption, production of bioethanol is very low. Between 2010 and 2011, the national capacity for ethanol production was 440 million liters, which is equivalent to about 1% of total liquid fuel consumption (Biofuel Association of Australia, 2012). The Australian government does not mandate minimum levels of inclusion of biofuels in petrol or diesel (Utilities Science and Innovation Committee, 2015), although some states mandate relatively low levels of ethanol in petroleum (e.g., Queensland’s Liquid Fuel Supply [Ethanol and Other Biofuels Mandate] Amendment Act 2015 and New South Wales’ Biofuels Act 2007). The biofuels industries in Australia remain under pressure and many biofuel plants have ceased operations (Biofuel Association of Australia, 2016). In any case, it is clear that government support is important in the initial stages of developing biofuels industries around the world.

## Future Directions

Despite the current slump in international oil prices, the ongoing volatility and political manipulation of these fossil fuel markets and the rapid recent rise of electrical technologies for cars, trucks and home heating, there is increasing acknowledgment that the global introduction of renewable liquid transport fuels remains of the highest priority for the future of planet Earth, which is suffering serious adverse reactions to elevated atmospheric CO_2_ concentrations. The current multidisciplinary approach to the challenges outlined in this short review will undoubtedly continue, with attention focused on methods for improving the composition of plant cell walls where crop residues and specialist plant species are used as biomass sources. Genetic engineering of biofuel crop species is unlikely to encounter the level of consumer resistance experienced with the attempted introduction of various GM food crops. Attention will also remain focused on the alleviation of the recalcitrance of plant material to the release of fermentable sugars and other valuable wall degradation products, and simultaneously on the development of well adapted microorganisms for the efficient fermentation of monosaccharides released from the cell walls of various feedstocks. Finally, the rapid development of more generic conversion technologies that will handle a wide range of quite different feedstocks without extensive or time-consuming modifications can be considered as one of the highest priorities for the conversion of plant cell wall residues to renewable liquid transport biofuels.

## Author Contributions

H-TT, KC, and GF conducted literature review and wrote the manuscript.

## Conflict of Interest Statement

The authors declare that the research was conducted in the absence of any commercial or financial relationships that could be construed as a potential conflict of interest.
